# A Critique of Helsinki Criteria for Using Lung Fiber Levels to Determine Causation in Mesothelioma Cases

**DOI:** 10.5334/aogh.3135

**Published:** 2021-07-29

**Authors:** Triet Tran, David Egilman, Mark Rigler, Theresa Emory

**Affiliations:** 1 Never Again Consulting, Attleboro, MA, US; 2 Clinical Professor of Family Medicine, Alpert School of Medicine Brown University, US; 3 ASPEX, LLC, Lawrenceville, GA, US; 4 Peninsula Pathology Associates, Newport News, Virginia, US

## Abstract

Asbestos is a known human carcinogen and the chief known cause of mesothelioma. In 1997, a group of experts developed the Helsinki Criteria, which established criteria for attribution of mesothelioma to asbestos. The criteria include two methods for causation attribution: 1) a history of significant occupational, domestic, or environmental exposure and/or 2) pathologic evidence of exposure to asbestos. In 2014, the Helsinki Criteria were updated, and these attribution criteria were not changed. However, since the Helsinki Criteria were first released in 1997, some pathologists, cell biologists, and others have claimed that a history of exposure cannot establish causation unless the lung asbestos fiber burden exceeds “the background range for the laboratory in question to attribute mesothelioma cases to exposure to asbestos.” This practice ignores the impact on fiber burden of clearance/translocation over time, which in part is why the Helsinki Criteria concluded that a history of exposure to asbestos was independently sufficient to attribute causation to asbestos.

After reviewing the Helsinki Criteria, we conclude that their methodology is fatally flawed because a quantitative assessment of a background lung tissue fiber level cannot be established. The flaws of the Helsinki Criteria are both technical and substantive. The 1995 paper that served as the scientific basis for establishing background levels used inconsistent methods to determine exposures in controls and cases. In addition, historic controls cannot be used to establish background fiber levels for current cases because ambient exposures to asbestos have decreased over time and control cases pre-date current cases by decades. The use of scanning electron microscope (SEM) compounded the non-compatibility problem; the applied SEM cannot distinguish talc from anthophyllite because it cannot perform selected area electron diffraction, which is a crucial identifier in ATEM for distinguishing the difference between serpentine asbestos, amphibole asbestos, and talc.

## Background

Using the Helsinki Criteria, a number of microscopists count asbestos bodies and fibers found in the lungs at autopsy of cases where asbestos exposure to a specific source cannot be determined and refer to this as background level. They compare these levels to fiber counts in workers or bystanders who have a known or suspected exposure to asbestos released from a known or suspect source. All the exposed cases have known or suspected exposures because all of them have made claims for compensation. These same microscopists speculate that these background levels are always lower than the fiber counts of exposed-caused cases. As a result, pathologists claim they can rule out these alleged exposures as a cause of mesothelioma if lung fiber/ferruginous body levels are below the background levels in historical controls [1].

Landrigan and colleagues [2] and the Collegium Ramazzini [2] had previously criticized the Helsinki Criteria [2,3]. The Helsinki Criteria committee responded that its report did “not make recommendations about methods for asbestos fiber analysis [4]”. Furthermore, the Helsinki group admitted the pathologic criteria are based on an unevaluated laboratory method. However, the Helsinki group has naturally endorsed that laboratory’s methods by basing the committee’s pathologic criteria on the laboratory’s fiber analysis. That laboratory has produced data and methodology for these key publications as part of discovery in asbestos litigation where one of the authors (Roggli) served as a witness for asbestos product manufacturing companies. As part of the legal process, one of us (MR) was allowed to inspect the laboratory and observe the methodology.

In its response, the Helsinki committee claimed that “the criteria do in fact consider work histories as the pre-eminent way of establishing asbestos exposure.” However, in litigation, pathologists have ignored this admonition and asserted that fiber methods should be used as the sole criteria for attribution of causation. We analyzed newly produced information to reassess the evidence and validity of using lung fiber count as a sole criterion for attribution of causation. In addition, we reviewed recent efforts by the same group to extend the Helsinki Criteria to the attribution of fibrous talc as a cause of mesothelioma [5].

## Methods

We reviewed the methodology used to establish background exposures in the 1997 and 2014 Helsinki Criteria, which were adapted from three publications from a single laboratory [1,6,7]. We used the key 1995 paper by Srebro and colleagues to investigate the influence of patient history and routine and electron microscopic examination on the ability to use fiber counts as a predictor of background exposure and causation of mesothelioma [1]. We reviewed relevant depositions and laboratory records of mesothelioma cases and controls from the cases reported in this publication to acquire information on histories and laboratory procedures that was not available in published papers. Additionally, we reviewed the recent application of one of the 1995 background controls in a publication of talc and mesothelioma.

## Results

### Analysis of the Helsinki Criteria’s methodology

#### 1. Background Exposure Sources

Most population exposure originates directly or indirectly from asbestos released from products during installation and removal [8]. Workers and bystanders are also exposed in product manufacturing and mines [9]. In cities, indirect exposures have occurred during the use of sprayed asbestos insulation and from the release of asbestos from asbestos containing products, including asbestos brakes [10]. Asbestos has been used in over 3000 products and is found as an accessory mineral in other commercially utilized minerals. Most bystanders and some product users are unaware of the fact that they were exposed to asbestos. Although many authors mention background fiber levels, neither the Helsinki Criteria nor any published literature clarify the source(s) of this background exposure. Thus, background is an exposure of exclusion dependent on the quality of the history and the patient’s knowledge of previous sources of exposure. In the absence of an identifiable history of exposure to an asbestos source, researchers appear to assume that asbestos fibers in lung tissue stemmed from exposure to asbestos in ambient air [11]. However, except in rare circumstances (e.g., the Grand Canyon), ambient exposures result from product use. In most cases, lung asbestos levels are merely comparisons of exposures to various commercial sources of asbestos. As a result, the identification of past sources of exposure is the Achilles heel of the definition of background exposure.

#### 2. Asbestos and lung fiber levels

Srebro and colleagues [1] described the use of lung fiber counts as a method for attributing causation to asbestos exposure and used this set of controls to distinguish fiber levels that result from background exposures from occupational exposures [1]. If a mesothelioma case’s fiber burden is below this background, the authors concluded that the mesothelioma was “spontaneous” or “idiopathic and unrelated to asbestos fibers found in the patient’s lungs [1]”.

#### 3. Inadequate control occupational history

Initially, Srebro and colleagues selected twenty patients from available pathology specimens at a VA hospital, which enrolled patients to serve as controls [1]. The authors defined their controls as “cases with no documented history of asbestos exposure” in the medical records and “no evidence of asbestos-related disease” in lung tissue [1]. However, the authors found high amosite fiber levels in one patient who was initially assigned to the controls. In response, they conducted “an extensive search through this patient’s medical records and [made] two phone calls to surviving relatives [which] revealed that his employment history included installing furnaces, an occupation associated with asbestos exposure [1]”. They then excluded this case from their analysis. Thus, their original screening missed important occupational exposures and classified a case with asbestos product exposure as an unexposed control.

Despite the fact that this case revealed the inadequacy of their screening, Srebro and colleagues failed to apply this “extensive search” to any of the other controls [1]. This is a cause of concern, because based on medical records alone, at least thirteen of the controls worked in occupations with potential asbestos exposure. These jobs include manual labor, Air Force, Truck driver, Garage owner, Spinning mill, Electrical engineer, and Hospital aide [1]. It is important to note that the authors only identified one job per control and failed to acquire a complete occupational history. The authors also had no information on smoking for 10 of the controls, indicating that they either did not have access to complete medical records or that the histories were insufficient for analysis [1]. Srebro, a medical student, collected and analyzed the history data [12]. She had no training in occupational medicine and did not use a standard questionnaire to evaluate the exposure histories [12].

#### 4. No basis for use of asbestos bodies to determine exposure levels

Had Srebro and colleagues included the furnace installer in their analysis, there would be no significant difference in asbestos bodies, total fibers, or chrysotile between cases and controls [13]. Albin and colleagues confirmed that one cannot make inferences about past asbestos exposures from lung fiber burden because “no quantitative differences in exposure (duration or intensity) could be shown between workers with high and low to intermediate [lung fiber burden] concentrations [14]”. Newman and colleagues also reported that “asbestos bodies or ferruginous bodies are nonspecific, because they can be found in occupationally unexposed individuals, in occupationally exposed individuals who have no asbestos-related lung disease, and in workers who have asbestosis [15]”. In addition, background and occupational asbestos exposures decreased during 1980–1995 due to increased occupational and environmental regulation and dramatic reduction in asbestos use [16,17]. It is unreasonable to compare background fiber levels in occupationally exposed plaintiffs who died in 2020 to controls who died 15–30 years earlier.

#### 5. Inappropriate statistical analysis

In addition, Srebro and colleagues inappropriately used a one-tailed Wilcoxon test to compare fiber levels. The authors should have performed a two-tailed test because 18/19 of the controls had higher fiber counts than the mesothelioma cases’ lowest count [13]. Using a two-tailed test, even excluding the outlier control case, the controls and cases fiber counts are not statistically significantly different.

#### 6. Potential control exposure

Srebro and colleagues’ controls were male veterans from states surrounding North Carolina who were examined from 1980 to 1995, while the cases were litigation referrals from the entire country [1,5,12]. These controls resided in North Carolina or surrounding states [18]. Much of the asbestos textile industry was based in North and South Carolina [19].

#### 7. No report of last exposure time for any case

Absent continuing exposure, lung fiber levels decrease over time as a function of fiber type and length [20,21,22]. Churg and colleagues reported a “significant negative correlation” between fiber concentration and time [23]. For cases, Srebro and colleagues did not report the date of last exposure prior to autopsy [1]. Thus, the same lung fiber count may reflect either a recent relatively low exposure in a patient who was exposed shortly before death or what remained from a much higher total exposure in another patient whose last exposure predated death by decades or anything in between.

Hence, fiber burden at death is a poor indicator of past exposure. Low fiber counts can be consistent with high exposures that occurred decades before death because fibers that are short and particularly chrysotile are not biopersistent in the lung [2,12]. Because asbestos translocates to the pleura, fibers may still cause or contribute to mesothelioma without being retained in the lungs. Conversely, recent exposures that do not contribute to disease causation may result in high fiber levels. Dr. Roggli, the co-author who performed the fiber analysis for Srebro and colleagues, has acknowledged that “fiber burden studies do not accurately reflect past exposures to chrysotile [24]”. Absent information on time from last exposure, the same is true for all fiber types, because pathologic evaluations cannot determine when a fiber entered the body. Therefore, low levels of lung fiber burden at death can be consistent with an elevated risk of mesothelioma.

#### 8. Incorrect assumption that asbestos body count correlates with total fiber exposure

Srebro and colleagues also relied on lung asbestos body counts (AB) to distinguish “exposed” and “idiopathic” mesothelioma patients by establishing an arbitrary background cut off [1]. If a patient’s asbestos body count was less than 20 AB/g wet lung, Srebro and colleagues classified the patient’s fiber lung burden as a control [1]. However, asbestos body lung count is not a robust indicator for fiber lung burden [25]. Chrysotile comprises 95% of all asbestos used in the US, but AB formation is far more likely to occur with amphiboles [8,26,27]. Aust and colleagues explained that “chrysotile is typically inhaled as a shorter, thinner particle form of asbestos and is much less likely to be found as the core of ferruginous bodies in human tissue than amphibole REMPs [respirable elongated mineral particles] [26]”. Thus AB counts always underestimate total fiber exposure. Churg and colleagues stated that counting of asbestos bodies “cannot be used to document total lung asbestos burden” because “the bulk of asbestos in these lungs was short chrysotile, which does not form bodies [28]”.

Besides, while uncoated fibers tend to concentrate in the lower lobes, Gylseth and colleagues reported that “the number of fibers coated [asbestos bodies] were generally higher in the upper lobes than in the lower ones [29,30]”. Another problem in using asbestos bodies as a benchmark for background exposure is the fact that the time required for formation of ferruginous bodies is not known, and individuals vary in their ability to deposit iron on fibers [26]. This is evident from Srebro and colleagues’ data, which showed that there was almost no relationship between ABs and uncoated fibers (R^2^ ranged from 0.0099 to 0.0769). Even the 1997 Helsinki Criteria concluded that “there is a poor correlation between asbestos body concentrations and chrysotile fibre burdens [31]”. Furthermore, Srebro and colleagues mistakenly claimed that their 19 controls were within the “norman range” of lung asbestos bodies (0–20 AB/g wet lung). One control (Case #19) had 22 AB/g [1].

#### 9. Problems with SEM analysis and fiber identification

Srebro and colleagues used SEM to detect and identify asbestos fibers in the lungs. However, the United States Environmental Protection Agency (EPA) concluded that SEM was inadequate for analysis of asbestos fibers because “SEM is limited in its ability to identify the crystalline structure of a particular fiber [32]”. SEM also lacks the ability to distinguish amosite and crocidolite, because SEM cannot easily detect sodium (Na) unless the investigators have a high resolution detector to pick up light elements [28].

In addition, SEM has limited ability to detect thin fibers [33]. In 1991, the Health Effects Institute (HEI) stated that SEM is “unsuitable for determination of asbestos fibers” because identification of a thin asbestos fiber required that “both resolution and contrast be sufficient [33]”. Because SEM has to sacrifice resolution for better contrast, SEM normally cannot detect fibers thinner than 0.2µm [33]. Dr. Roggli used 650X magnification power and not 1000X to identify fibers [1,13,34]. Roggli could not identify asbestos fibers thinner than 0.3 µm at 650X, and 98.7% of chrysotile fibers in the lung are less than 0.25 µm wide [34,35]. Due to these SEM limitations, Dodson and colleagues noted that transmission electron microscopy (TEM) is “the most accurate instrument for detecting and analyzing asbestos fiber types in a sample and appropriately providing their dimensions [36]”. Roggli and colleagues observed that “fiber counts are usually 3-fold higher with TEM than with scanning electron microscopy [37]”. All other US agency protocols that relate to fiber counting rely on TEM analysis, which provides morphology, chemistry by EDS, and crystallinity by selected area electron diffraction (SAED) [12].

Srebro and colleagues claimed to count all fibers that were longer than 5 µm with an aspect ratio of 3:1 or higher [1] However, they did not document the fiber length measurements. They counted fibers that were visually estimated to be longer than 5 microns and included fibers with detectable diameters seen at lower magnification in the SEM. Visually estimating fibers longer than 5 µm is an unreliable method, which often underestimate asbestos count [38]. They also “did not record and retain a photographic record of any of the fibrous structures encountered [34]”. Short fibers (less than 5 µm) have been shown to be carcinogenic and are concentrated in the pleura [3,39].

In addition, Roggli did not employ appropriate filters. Srebro and colleagues used 0.4-µm pore size polycarbonate filters to collect digested lung tissue [1]. Sullivan and colleagues reported that 88% of the fibers had diameters smaller than the 0.4-µm pore size, which could cause “unacceptable losses” of fibers during investigations using electron microscopy [40]. Thus, a significant number of small asbestos fibers in the lungs may have passed through filters used by Srebro and colleagues.

On the other hand, Srebro and colleagues did not employ geologic criteria to differentiate fibers originating from an asbestiform habit from cleavage fragments (fiber population, habit, parallel fibers occurring in bundles, fiber bundles displaying splayed ends, matted masses of individual fibers, and/or fibers showing curvature) [1,41]. We agree that fiber formation origin does not reflect carcinogenicity; however, this view is not universally accepted [39,41].

#### 10. SEM and talc

SEM cannot usually distinguish anthophyllite from talc [42]. Srebro and colleagues assumed that any iron free fiber with MgSi peaks identified by SEM was talc and did not report anthophyllite in any case or control [1]. This is not the case [42,43]. The International Organization for Standardization (ISO) noted that SEM cannot routinely discriminate anthophyllite with a low iron concentration from talc with a high iron concentration [42]. ISO noted, “The fibre morphology can assist [SEM] in discrimination between anthophyllite and [non-fibrous] talc. Ribbon-like fibres are probably talc, whereas straight, rod-like fibres are possibly, but not necessarily, anthophyllite [42]”. Dr. Roggli noted that the fibers he designated as talc were straight and rod shaped. Thus, they were indistinguishable from anthophyllite [44].

ISO remarked that talc should be evaluated using TEM [42]. The Bureau of Mines supported this recommendation: “It is when fibrous amphiboles occur with fibrous talc that morphology alone is inadequate to distinguish between phases. For this reason, TEM has been recommended for regulatory use [45]”. Srebro and colleagues could also have misidentified chrysotile as talc, because they did not do SAED and could not determine if serpentine, amphibole, or talc crystalline patterns existed for the elongated mineral particles [36]. Srebro and colleagues also could not tell if a talc fiber was not a bundle of chrysotile, because SEM at low magnification cannot always identify fibrils [36]. Additionally, chrysotile has a Mg-Si ratio of 1:1 but preferentially loses Mg in-vivo through biodegradation [21,22] However, Srebro and Roggli classified fibers with 2:3 MgSi ratios as talc [34]. Roggli claimed he used morphology to distinguish talc from chrysotile [46]. However, short chrysotile fibers (less than 10 µm) are often straight and look similar to talc when observed by SEM [47,48,49]. Therefore, Srebro and colleagues may have conflated chrysotile with talc, undercounting chrysotile and overcounting talc [50].

#### 11. Problems with consistency and reproducibility

Fiber counts are not reproducible or consistent, because fibers are not evenly distributed in the lung lobes, sample sizes vary, and investigators do not provide data on the location of the samples in either controls or cases. Using a background fiber level as a universal cut-off to determine past asbestos exposure relies on two assumptions: the intralaboratory results are consistent, and the background fiber levels are geographically uniform. However, in the real world, neither of these assumptions is true. Contrary to Srebro and colleagues’ claims, there is no standard background exposure, because background asbestos lung levels are related to geographic location, decade of exposure, and local industries (factories, shipyards, or asbestos product manufacturing plants) [8]. In addition, Srebro and colleagues did not collect any information on potential bystander exposure for controls and never evaluated this potential confounder [12]. There were no occupational histories of spouses, siblings, parents, or residential history for controls. The levels of indirect asbestos exposure vary greatly from location to location and have dropped over time [8]. Roggli and colleagues reported highly variable fiber concentrations in the air samples of 48 US cities [6]. The Agency for Toxic Substances and Disease Registry (ATSDR) documented remarkably different background levels from asbestos facilities across different U.S. states (0–1371 pounds per year in 1999) [8]. The ATSDR concluded that using lung fiber burden as an indicator of occupational asbestos exposure could lead to “false negatives” due to

analytical variability due to contamination or loss in processing, variability in retention of fibers in different regions of the lung and variability in sampling of different lung regions, variability in exposure parameters including fiber type, length, and width, and variability in individuals’ physiological parameters influencing retention [8].

Due to SEM limitations, the reproducibility of fiber levels in human lungs is poor. Feder and colleagues found an 8.5-fold increase in human lung fiber burden after 22 years of cessation from asbestos exposure [51]. Roggli noted that the lung fiber burden of two samples taken from the same patient can differ by as much as a factor of one thousand [52]. Oury and colleagues noted, “Interlaboratory comparison trials demonstrate that striking differences can occur among laboratories even when the same sample is analyzed” due to changes in a laboratory’s procedures or to variation of fiber burden level from one site to another within the lung [53]. Gylseth and colleagues reported that it is difficult and inappropriate to directly compare tissue burden analysis results from one laboratory to another [54]. The ATSDR stated that “asbestos contamination of laboratory materials, including paraffin, grids, and especially cross-contamination by tissue themselves must be accounted for [8]”. For these reasons, Case and colleagues noted that “laboratories that do this type of work [fiber burden analysis] should, therefore, have good control values [55]”. However, Srebro and colleagues did not use positive or negative controls [50].

Churg and colleagues reported a statistically significant difference between numbers of fibers in central versus peripheral lung [28]. Anttila and colleagues stated that “there was a greater number of 3 µm and longer fibers in the lung tissue of patients who had a lower-lobe tumor as compared with those who had an upper lobe tumor (Student’s t-test, p < 0.01) [29]”. Churg and colleagues observed that “fibre concentration tended to be greatest at the apex of the lung, whereas the longest and highest aspect ratio fibres were seen in the lower zones [30,56]”. In the face of this variability, using the maximum fiber count as the background level is not reasonable. Applying a 2–3-fold range to fibers for the control or case counts will often convert the interpretation of a case from idiopathic to exposure-related or vice versa.

### Application of Helsinki Criteria to fibrous talc assessed as lung fiber levels

There is no background source of exposure to talc. Pavlisko and colleagues and Roggli and colleagues relied on Srebro and colleagues’ controls to establish background lung concentrations for talc despite the fact that none of these papers collected information on either industrial or consumer talc exposure for either cases or controls [5,57]. In particular, Pavlisko and colleagues stated, “Talc is a nonasbestos mineral fiber that is considered elevated above background range at 10,000 fibers per gram.” However, unlike asbestos, talc exposure has never been measured in any background or environmental setting. While talc was used in wall board, paint, food (chewing gum), paper, drug expedient, and rubber manufacturing, there is no evidence that any of these uses result in airborne exposures to non-workers. The most common exposure to fibrous talc occurs during use of cosmetic talc powders [58,59,60].

Out of Srebro and colleagues’ 20 available controls, Pavlisko and colleagues selected a single control case (#24) with the highest talc fiber level as the background level for lung talc [1,5]. The use of this case is problematic. Case #24 was a male military veteran who died from Alzheimer’s disease and whose job history was unknown and unobtainable. Control case #24 had a combined tremolite, actinolite, and anthophyllite (TAA) level that was higher than 7/18 mesothelioma cases [1]. Case #24’s NAMF level (10,160 f/gm), which was extrapolated by Roggli to be all talc fibers, is higher than that found in 88% of the mesothelioma cases in Srebro and colleagues [1].

Roggli admitted that the lung fiber levels in Srebro and colleagues’ controls were not a “balanced representation” of fiber types [61]. Srebro and colleagues only analyzed fiber type for 5 out of 26 fibers counted in controls and extrapolated the rest. Srebro and colleagues used Case #24 to establish background talc exposure but only analyzed fiber type for 5 of 26 NAMF [62]. After those 5 fibers were identified, the ratio of those fiber types was applied to the remaining 21 NAMF fibers counted [62]. Using this extrapolation methodology, the total talc fibers of Srebro and colleagues’ control cases ranged from 0 to 10,160 f/gm. As a result, Roggli’s statistical inference of talc background fiber is prone to significant random sampling errors, because he did not investigate all available data (5 fibers are unlikely to be representative of 26 fibers) [62].

Srebro and colleagues’ post hoc rejection of a control case (the furnace worker) is evidence that outliers more likely represented a failure to obtain a history of past exposure rather than a true background level. The controls had a mean talc count of 981.8 f/gm with a standard deviation of 2,354 f/gm. Case #24’s talc fiber level, 10,160 f/gm, is almost four standard deviations higher than the average for the control cases. Thus, Roggli and colleagues and Pavlisko and colleagues used an outlier to establish the talc background for all control cases [5,57]. Srebro and colleagues did not consider geographic location or potential exposures to household members who may have worked with asbestos or talc [1,28]. Notably, most users are unaware of the fact that talc-based baby powders contain asbestos, and adults are often unaware of their own exposure to baby powder as infants. Talc manufacturers admit that cosmetic talc contained asbestos until the early 1980s [63]. If fiber burden is to be used for attribution of causation, case fiber levels should be compared to the lowest background count or the average count, especially when control exposures are based on postmortem medical records where histories do not include any specific evaluation of possible consumer, occupational, familial, or bystander exposure to asbestos or talc.

Roggli and Pavlisko selected the highest talc fiber lung burden among the controls to establish the 10,000 fibers per gram background. Roggli relied on the Helsinki Criteria to justify counting the highest fiber level rather than the average level as background for talc lung content [61]. Roggli explained, “Helsinki requires that the asbestos content be above the range of values for a background population [61]”. However, Roggli misinterpreted Helsinki by conflating necessity and sufficiency. The 1997 Helsinki criteria stated, “A lung fiber count exceeding the background range for the laboratory in question… should be *sufficient* to relate a case of pleural mesothelioma to asbestos exposure on a probability basis” [emphasis added] [64]. Helsinki does not indicate that an asbestos lung fiber count above background range is *necessary* to attribute causation to asbestos exposure [64]. Helsinki does not indicate that a lung fiber count below background range rules out asbestos as a cause of mesothelioma. In fact, it explicitly recognizes that a history of even low exposure is sufficient to establish a causal nexus [64]. In addition, the Helsinki Criteria focused on asbestos and did not mention talc, asbestiform talc, or other asbestiform structures [64].

Despite the technical issues noted above and the fact that they did not take any occupational or consumer product use history, Roggli and colleagues and Pavlisko and colleagues used talc fiber levels found in control Case 24 as a background fiber burden for talc [5]. If a patient’s talc fiber level is less than Case 24’s 10,000 fibers per gram, Roggli would classify the mesothelioma to be idiopathic and unrelated to any talc exposure [57]. This is inconsistent with Roggli’s observations of tremolite/actinolite and anthophyllite together with fibrous talc in the tissue samples. In the absence of chrysotile, industrial or cosmetic talc exposure is the most likely source of the tremolite/actinolite and anthophyllite [60]. Roggli and colleagues confirmed that fibrous talc in tissue is “correlated strongly” with tremolite in tissue and both are “derived” from talc exposure [65]. Talc is the only commercial product that has been reported to contain the combination of tremolite and/or actinolite and/or anthophyllite. Actinolite is not present in any commercial product apart from talc. Thus, the talc exposure is unlikely to be a result of background exposure [60]. Johnson & Johnson estimated that Johnson’s Baby Powder had 70% market share and that by 1992 this product had been used on 200 million “baby bottoms,” exposing both the parents/caregivers as well as the babies to inhalation of talc and other particulates that are in the talc [66]. Cosmetic talc body powder exposures can be quite high during normal use and have resulted in fatal cases of talcosis [67]. Talc has been used as a dry shampoo, in various body and perineal applications, and in talc-based animal flea powders. Therefore, we believe that any lung burden of talc in combination with tremolite, actinolite, or anthophyllite asbestos is most likely a result of exposure to cosmetic talc and not from ambient air. In a deposition, Roggli noted, “The cases we have done fiber analysis on have not shown—have not analyzed whether an individual was exposed to cosmetic talc or not. So certainly I cannot use my database experience to comment upon exposures from cosmetic talc [68]”.

## Conclusion

The Helsinki pathologic criteria for attribution of asbestos causation to mesothelioma were based on Srebro and colleagues’ work. For this paper, we analyzed the data and methodology that was more fully explained in court documents. We conclude that the method used to determine background lung fiber levels is fatally flawed and that quantitative analysis of a background level of asbestos exposure cannot be established by tissue analysis as performed and that occupational histories for controls do not reliably rule out the absence of exposure to asbestos released from commercial products. Srebro and colleagues proved that the medical records initially resulted in misclassification of at least one patient, who on more detailed evaluation was believed to have been occupationally exposed to asbestos. Despite this, Srebro and colleagues failed to obtain a more detailed evaluation of any other control, including a patient who had Alzheimer’s disease and was thus incapable of providing any meaningful occupational history. We believe that lung fiber burden cannot rule out significant previous exposure to asbestos mesothelioma causation compared to background fiber counts due to

1. the lack of biopersistence in the lung but not the pleura combined with the lack of matching on year of last exposure,
2. variability of fiber levels in different parts of the lung,
3. sample size variability,
4. intra-laboratory variability.

The methodology employed by Srebro and colleagues lacked both statistical reliability and microscopic precision. Due to the many confounders inherent to lung fiber burden, a detailed and correct occupational history is still the best tool to determine if a case of mesothelioma is related to asbestos exposure. Further, using historical background controls from Srebro and colleagues’ paper to establish background talc is inappropriate. Pavlisko and colleagues have not established that there is any non-product background exposure for talc. Bystander exposure to talc product use or to users of cosmetic talc products cannot be considered background. The authors admit that they never considered any history of talc exposure, neither did they record the presence of platy talc in any controls or mesothelioma cases.

Lung fiber burden is useful to determine the type of fibers a patient is exposed to, but its quantitative analysis of a background level is inadequate and misleading when used as the only tool in risk assessment or in the attribution of asbestos causation. There is no known threshold asbestos dose that does not increase the risk of contracting mesothelioma. Even if a threshold exists, there is no way to correlate lung fiber levels to any risk threshold.
